# Analysis of Meiotic Behavior and 2n Pollen Formation Frequency in Triploid Hybrids of Chinese Jujube

**DOI:** 10.3390/plants14111643

**Published:** 2025-05-28

**Authors:** Yunxi Zhong, Lixin Ge, Yinfang Song, Zhi Luo, Jiurui Wang, Mengjun Liu, Fenfen Yan

**Affiliations:** 1 The National-Local Joint Engineering Laboratory of High Efficiency and Superior-Quality Cultivation and Fruit Deep Processing Technology on Characteristic Fruit Trees, College of Horticulture and Forestry, Tarim University, Alar 843300, China; yunxixi200004@163.com (Y.Z.); 15128637300@163.com (L.G.); 17399978647@163.com (Y.S.); 15930225993@126.com (Z.L.); 2 College of Forestry, Hebei Agricultural University, Baoding 071001, China; wjrjujube@126.com; 3 College of Horticulture, Hebei Agricultural University, Baoding 071001, China; lmj1234567@aliyun.com

**Keywords:** Chinese jujube (*Ziziphus jujuba* Mill.), triploid, hybrid progeny, 2n pollen, meiosis, germplasm

## Abstract

The Chinese jujube (*Ziziphus jujuba* Mill.), an economically significant fruit tree native to China, is valued for both fresh and dried uses. In plants, 2n gametes serve as the fundamental basis for creating a sexual polyploid germplasm. This study investigated the 2n gametogenesis frequency in triploid hybrid jujubes through meiotic analysis of the hybrid strain Q161 and a two-year pollen analysis on hybrid progeny, assessing the natural 2n pollen frequencies to identify a high-2n-pollen germplasm and revealing the occurrence of 2n pollen. Meiotic analysis of the triploid hybrid Q161 (2n = 36) revealed cytological anomalies, including binucleate cells (22.80% abnormal tetrads), with natural 2n pollen production rates reaching 4.00% and 4.67% over two consecutive years. Scanning electron microscopy (SEM) revealed that the 2n pollen exhibited pronounced exine ornamentation with cerebroid sculpturing and tubercle-like structures at the apertures. Analysis of the triploid progeny for two consecutive years demonstrated a pollen viability of 30.45% and 23.83% (CV: 19. 39–29.69%), with the mean 2n pollen frequencies of 22.52% and 7.64%, peaking at 52.16% and 28.95% in elite individuals. Six triploid germplasm accessions with naturally elevated 2n pollen frequencies were identified. Under natural conditions, a triploid hybrid germplasm in Chinese jujube produces 2n pollen grains due to abnormal meiotic behavior, and a natural triploid germplasm with high pollen productivity was identified. This research provides a critical theoretical foundation for sexual polyploid breeding strategies.

## 1. Introduction

The Chinese jujube (*Ziziphus jujuba* Mill.), a unique and economically significant fruit tree endemic to China, is rich in nutrients and holds substantial agricultural value. It accounts for 98% of the global cultivation area and yield. Notably, jujube fruits from Xinjiang exhibit exceptional quality due to the region’s distinctive environmental conditions [1,2,3,4]. However, conventional diploid breeding faces genetic limitations in improving fruit development and stress tolerance. To address this, researchers have developed polyploid germplasms through natural selection and artificial induction, which effectively enhances fruit quality [5,6]. At present, successful applications of polyploid breeding have been reported in kiwifruit [7], citrus [8], and pear [9]. However, the jujube itself is difficult to crossbreed and there are fewer reports on jujube polyploid breeding, so the process of jujube ploidy breeding and new variety selection is relatively slow and lagging behind, and the creation efficiency is low. As 2n pollen serves as the foundation for polyploid breeding, the development of a polyploid germplasm through 2n-pollen-mediated ploidy manipulation presents a promising strategy to address the challenges in jujube cultivar selection and industrial advancement. This approach has emerged as a pivotal pathway in Chinese jujube breeding programs, offering novel solutions to enhance genetic diversity and agronomic performance.

Meiotic irregularities are critical mechanisms underlying 2n gamete formation. For instance, in 2n pollen production in *Begonia*, there was abnormal segregation of the chromosomes during meiosis (e.g., chromosomes did not segregate during meiosis I but only during meiosis II) [10]. Additionally, chromosome stickiness and spindle misalignment can lead to 2n pollen formation [11]. Similar phenomena, such as spindle misorientation and chromosome stickiness during meiosis, have been repeatedly observed in *Populus*, resulting in diploid and triploid pollen grains [12]. In *Ranunculus auricomus*, chromosome stickiness during meiosis contributes to giant pollen grains and other abnormalities [13]. Collectively, these studies demonstrate that meiotic anomalies frequently induce 2n pollen production. Intriguingly, the generation of 2n pollen or gametes has been recognized as a predominant mechanism for polyploid formation. In contrast to somatic polyploidization, the development of polyploids via 2n gametes not only combines heterosis arising from hybridization but also integrates gene dosage effects, thereby significantly enhancing genetic diversity within polyploid breeding systems [14]. Researchers have extensively explored 2n gamete induction through temperature or chemical treatments in *Arabidopsis* [15], lily [16], *Camellia oleifera* [17], and longan [18].

Chinese jujube, a woody perennial species, exhibits significantly higher genetic heterozygosity compared to model plants such as *Arabidopsis thaliana*, and possesses a complex genomic architecture characterized by extensive structural variations and allelic diversity [19,20]. Current studies on meiosis in diploid and certain polyploid jujube genotypes have been documented [21,22]. However, the 2n gamete formation remain unexplored. Concurrently, 2n gametes in diploid jujubes exhibit low spontaneous occurrence rates, necessitating artificial induction (e.g., via somatic chromosome doubling or chemical treatments) to enhance their production efficiency [23]. The natural occurrence of 2n pollen in polyploid citrus has been documented in previous studies [24]. However, research on the natural occurrence of 2n gametes in triploid jujubes and their application in breeding programs remains limited. The creation of a triploid jujube germplasm remains technically challenging. Thus, exploiting naturally produced 2n gametes offers a promising avenue for developing elite polyploid germplasms.

This study employed triploid F1 hybrids derived from crosses between the diploid ‘Dongzao’ and tetraploid ‘Chenguang’ jujube varieties. We analyzed meiotic behavior in the triploid hybrids, identifying the spontaneous formation of 2n pollen, which under natural conditions was found to be associated with abnormalities during meiosis. Through biennial evaluations of the natural 2n pollen frequencies in the hybrid progeny, we screened multiple accessions with high 2n pollen yields and investigated the genetic variation characteristics of 2n pollen within populations. These findings establish a foundation for utilizing natural 2n gametes in jujube breeding. Future research will explore more effective methods to break through sexual polyploid germplasm innovation.

## 2. Results

### 2.1. The Analysis of Meiosis and the Abnormal Behavior of Pollen Mother Cells of the Triploid Germplasm

#### 2.1.1. Correspondence Between Flower Bud Size and Meiotic Period

**Figure 1:**
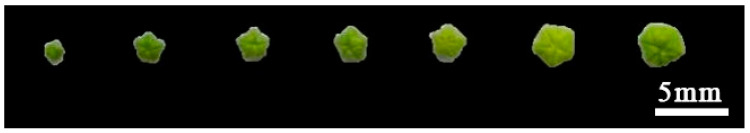
Flower bud diameter during meiosis of the jujube triploid superior line Q161.

By measuring the size, color, and sepal characteristics of the jujube date flower buds, the meiotic processes of the pollen mother cells in each period were observed using staining. Correspondence was found between the size, color, and the degree of sepal calyx cleavage of the flower buds and the meiotic period of the microspore mother cells (Table 1, Figure 1). Among them, flower buds of 1.3–2.1 mm in size were in the pollen mother cell period of Q161, when the color of the buds was dark green and the sepal lobes were not obvious. Subsequently, the flower buds entered the first meiotic division and remained dark green in color and slightly enlarged in size, with a diameter of 2.1 to 2.8 mm, which occurs mostly in prophase I of meiosis. The diameter of the flower buds ranged from 2.8 to 3.1 mm at the end of meiosis II, when the color of the flower buds appeared yellowish-green and the calyx cleft was more obvious, and the observation at this time clearly showed the different morphology of the tetrad of the microsporangial mother cells. Meiosis was completed when the diameter of the flower bud was 3.1–3.9 mm, at which time the sepals of the bud were about to split and yellow mature pollen grains could be observed. The correspondence between the flower bud size and meiotic period may provide a basis for later 2n pollen induction and pollen trait studies.

#### 2.1.2. The Process of Meiosis

**Figure 2:**
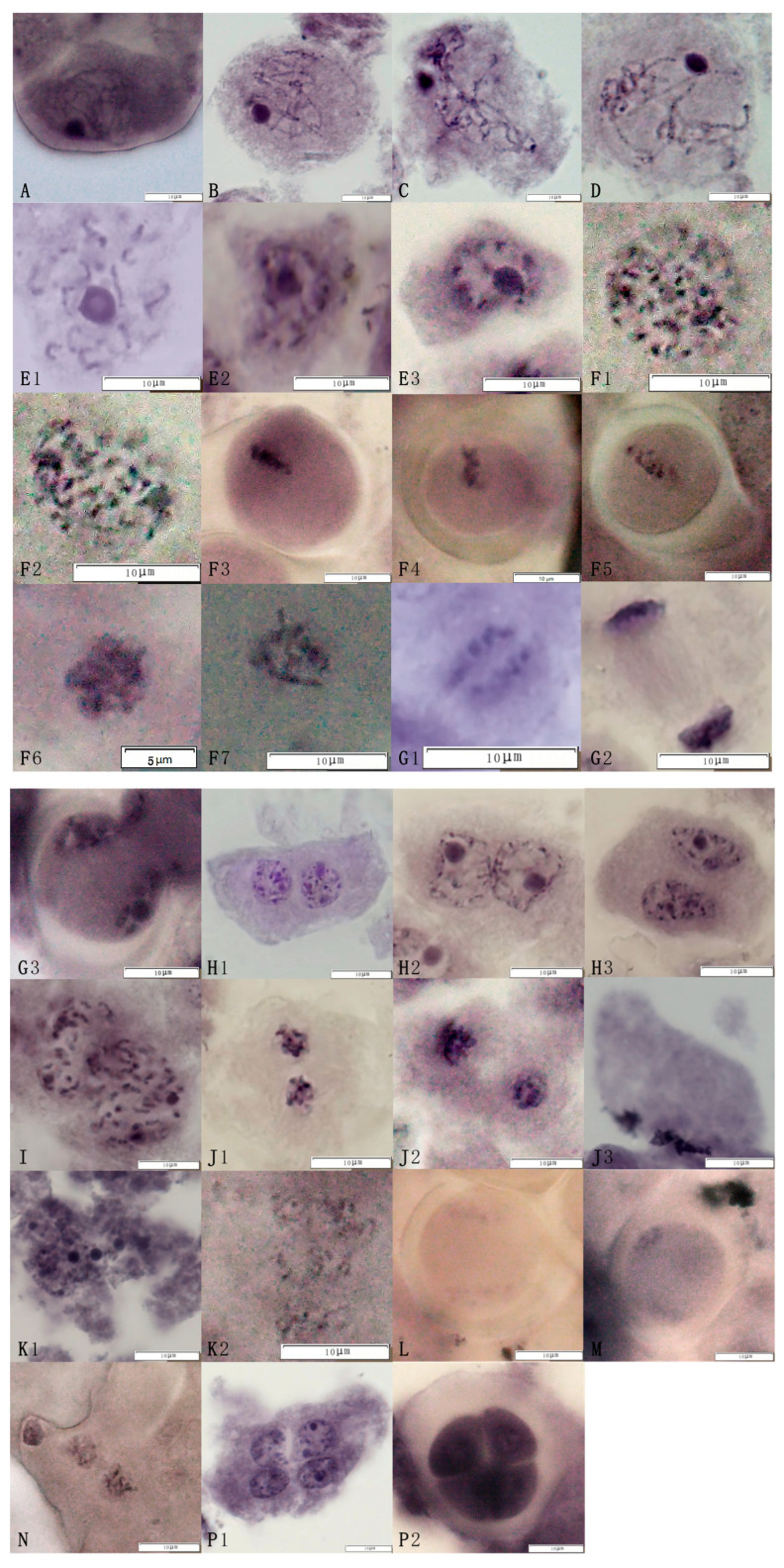
The meiosis process in pollen mother cells of the jujube triploid hybrid progeny Q161. Cells successively underwent the following phases: (**A**) Leptotene, (**B**) Zygotene, (**C**) Pachytene, (**D**) Diplotene, (**E1**–**E3**) Diakinesis, (**F1**–**F7**) Metaphase I, (**G1**–**G3**) Anaphase I, (**H1**–**H3**) Telophase I, (**I**) Prophase I, (**J1**–**J3**) Metaphase II, (**K1**,**K2**) Anaphase II, (**P1**) Telophase II, (**P2**) Tetrads.

The observations of meiosis in the pollen mother cells of the triploid euploid line Q161 showed that the meiotic process basically conformed to the pattern of diploid meiosis. The first meiotic division I (Figure 2A–D,E1–E3), the middle of meiotic division I (Figure 2F1–F7), the late meiotic division I (Figure 2G1–G3), and the end of meiotic division I (Figure 2H1–H3) were experienced in sequence. The cells then entered meiosis II and proceeded through meiotic prophase II (Figure 2I), meiotic mid-phase II (Figure 2J1–J3), meiotic late-phase II (Figure 2K1,K2), and meiotic terminal-phase II (Figure 2L–N), respectively. However, dichotomies (Figure 2L) and trichotomies (Figure 2M) appeared due to anomalous fusion. After meiosis I and meiosis II, the pollen mother cell divided to form tetrads (Figure 2P1,P2).

#### 2.1.3. Statistics of Abnormal Meiotic Behavior

**Figure 3:**
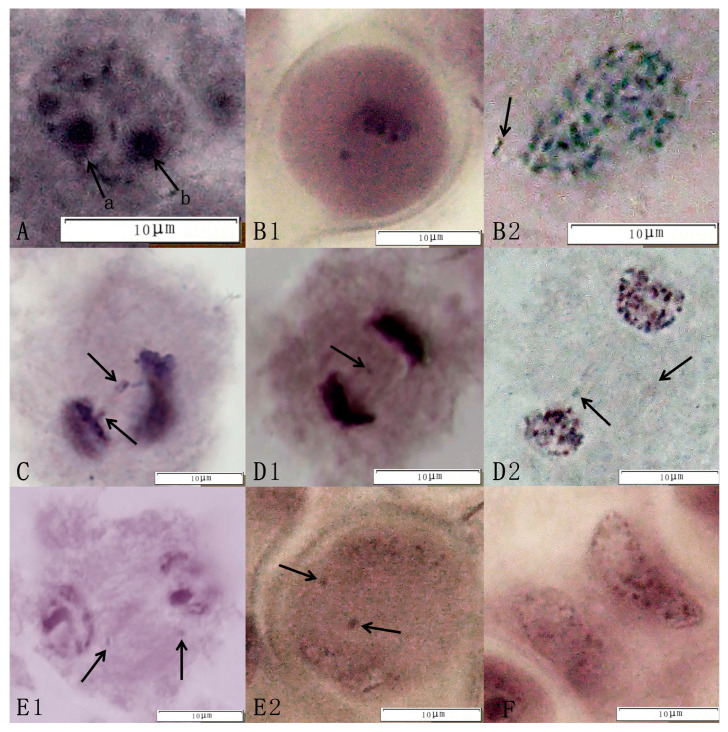
Abnormal meiotic behavior of the jujube triploid hybrid progeny Q161. Abnormal behavior included the following: (**A**) Diakinesis (arrows a and b in Figure 1 indicate double nucleoli of an equal size at Q161). (**B1**,**B2**) Metaphase I (arrows point to chromosomes outside the equatorial plate). (**C**) Anaphase I (showing unequal chromosomal segregation; arrows indicate chromosome bridges). (**D1**,**D2**) Anaphase I (arrows highlight lagging chromosomes). (**E1**,**E2**) Telophase I (arrows mark laggard chromosomes). (**F**) Anaphase II (showing premature cytokinesis).

The observation of meiosis in pollen mother cells of the triploid germplasm revealed multiple abnormal behaviors. For example, at the meiotic terminal metaphase, chromatin formed rods, “v” shapes, spheres, and other forms, i.e., monovalent, bivalent, and trivalent bodies (Figure 3A). At mid-meiotic I, one or more extra-equatorial plate chromosomes, the lagging chromosomes, appeared (Figure 3B1,B2). In chromosome movement, the unequal segregation of chromosomes and backward chromosomes appeared. In addition, the adhesion of chromosomes occurred, exhibiting the phenomenon of chromosome bridges (Figure 3C). In late meiosis I, homologous chromosomes separated from each other and moved toward the poles, resulting in two abnormal configurations, the parallel spindle and the perpendicular spindle (Figure 3D1,D2). At the end of meiosis I, chromosomes were separated by spindle filament traction toward the two levels, and new daughter nuclei appeared, but some cells showed extra nuclear chromosomes (Figure 3E1,E2). In late meiosis II, the cytoplasm divided early; there were chromosome bridges in the cell, and the chromosomes were unevenly segregated, which in turn led to the production of dichotomies and the formation of unreduced (2n) gametes (Figure 3F). In the present study, we found abnormal spindle traction, chromosome bridges, backward chromosomes, and unequal segregation during meiosis in the superior lines of hybrid progeny, all of which led to chromosome conformation complexity and ultimately led to abnormalities in the tetrad period and the emergence of different types of tetrasomes.

**Figure 4:**
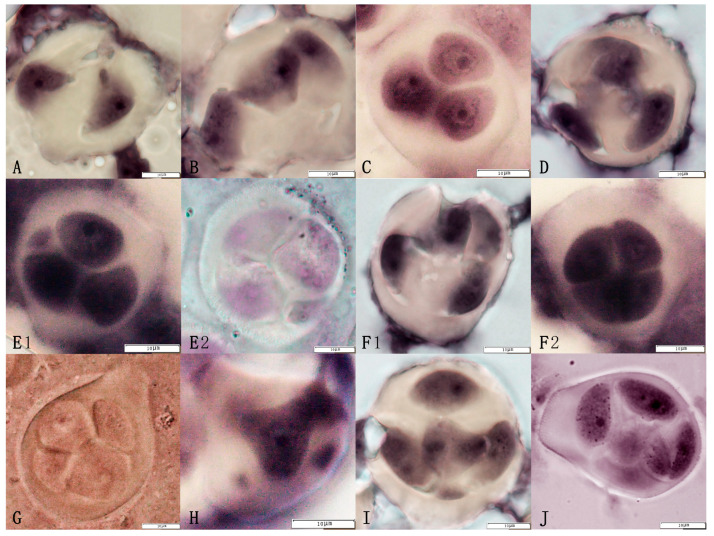
Abnormal tetrads in the jujube triploid hybrid progeny Q161. The abnormal tetrads included the following: (**A**) Dyad, (**B**) Trisomy, (**C**) Sequentially arranged trisomy, (**D**) Trisomy, (**E1**,**E2**) Trisomy + 1 micronucleus, (**F1**,**F2**) Tetrads, (**G**) Unequal division of the tetrads, (**H**) Pentads, (**I**) Pentameric + 1 micronucleus, (**J**) Hexads.

Observation of the tetrads formed by the triploid euploid line Q161 (Figure 4, Table 2) revealed the presence of several types, with 77.20% normal tetrads. In addition to the formation of normal tetrads, abnormal dividing tetrads such as the uneven separation of cytoplasm were observed. For example, a small number of dichotomies, trichotomies, pentamers, and hexamers were observed, some of which carried one or more micronuclei. The proportions of tetrads, trisomy, and dyads with micronuclei, among all the isolated types of cells, were 6.80%, 7.50%, and 4.60%, respectively. The triad with micronuclei, pentads with micronuclei, pentads, and hexads exhibited the lowest proportions, accounting for 1.90%, 0.30%, 1.30%, and 0.30%, respectively, with a total abnormality rate of 22.80% (Table 2).

#### 2.1.4. Determination of the Chromosome Number of Progeny Q161 of the Jujube Superior Line

**Figure 5:**
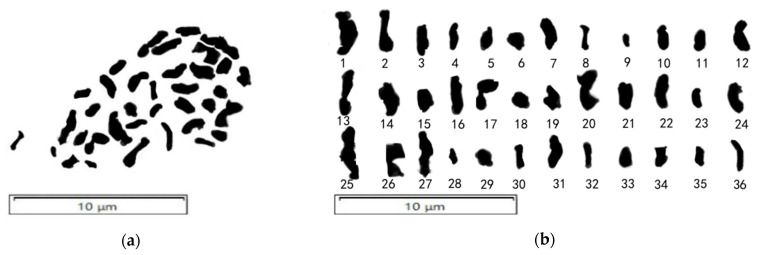
Number of metaphase I chromosomes of triploid elite line Q161 in jujube. (**a**) Whole chromosomes of germplasm Q161; (**b**) Chromosome counting results.

Cells of the triploid elite line Q161 at mid-meiotic I were selected for observation; the chromosome numbers were identified and the chromosome lengths were measured (Figure 5, Table 3). The chromosome morphology of Q161 was good; the number of chromosomes was 36 and the total length of the whole group of chromosomes was 62.771 μm. The chromosome lengths of Q161 were distributed between 0.828 μm and 3.058 μm (Table 3), with an average length of 1.744 μm, and the ratio of the longest chromosome to the shortest chromosome was 3.693.

### 2.2. Natural 2n Pollen Distribution of the Triploid Germplasm

Pollen traits of the triploid superior line Q161 were determined over two consecutive years (Table 4). Large differences were found between the pollen activity and pollen volume over the two years. The mean pollen diameter remained stable for two years. This superior line produced a high percentage of n pollen, 71.33% and 83.67% in each of the two years, respectively. The line was able to produce 2n pollen naturally and stably in both years, 4.00% and 4.67% in each of the two years, respectively. The stable production of 2n pollen has potential as parental material in polyploid breeding.

**Figure 6:**
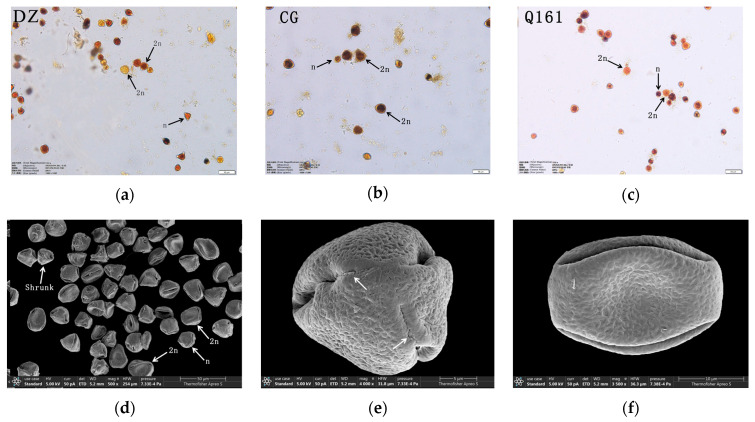
(**a**–**c**): Pollen morphology of DZ, CG, and Q161, respectively. Q161 electron microscope scanning observation: (**d**) Pollen whole, (**e**) Pollen side, (**f**) Pollen front.

The diameter and pollen morphology characteristics of triploid Q161 were further observed by scanning electron microscopy (Figure 6d–f). A large amount of pollen could be observed in the field of view, but more shrunk pollen (starch-deficient empty pollen) was present, which was consistent with the results of the pollen activity determination by I_2_-KI (Figure 6a–c). Electron microscope scanning images showed that the triploid zygote had more prominent outer wall ornamentation with a brain-like sculpture and obvious reticulation mesh, with only a small portion of the reticulation ridges at the extreme face end being parallel and cross-distributed; the germination pore was prominent and verrucose, and the top of the pollen was relatively flat.

### 2.3. Analysis of Pollen Traits in Jujube Triploid Hybrid Progeny Clusters

**Figure 7:**
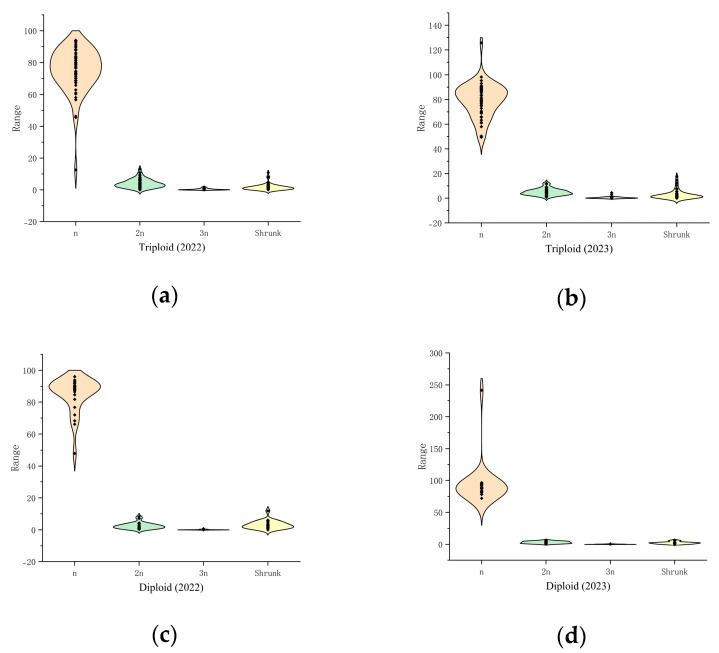
Frequency distribution of pollen types in several progeny groups. (**a**) Triploid offspring in 2022; (**b**) Triploid offspring in 2023; (**c**) Diploid offspring in 2022; (**d**) Diploid offspring in 2023.

This study of the two traits of pollen quantity and pollen activity in the triploid population of the hybrid progeny revealed additional details (Table 5, Figure 7). The triploid hybrid progeny had lower pollen activity and pollen volume than the diploid hybrid progeny cohort, with significant differences in 2023, and the pollen traits showed unstable performance between the two years. In 2022–2023, the pollen activity of the triploid hybrid progeny was 30.45% ± 9.04% and 23.83% ± 4.62%, respectively. The pollen counts were 942.53 grains ± 763.14 grains and 1438.96 grains ± 755.84 grains, respectively. It is conjectured that the pollen quantity was strongly influenced by environmental conditions, interannual variability, and genetic variation. It was also found that the pollen sizes differed. The difference between the pollen size and pollen activity of the parents and progeny can be clearly seen in Figure 7. Activity of the triploid hybrid progeny was lower than that of the tetraploid parent. The pollen of the triploid progeny was significantly smaller than that of the parent ‘Chenguang’ and larger than that of the diploid progeny.

The hybrid progeny were further analyzed for the magnitude of variation in pollen amount and pollen activity (Table 6). The coefficients of variation for the pollen activity of triploid hybrid progeny ranged from 19.39% to 29.69%, and those for the pollen quantity ranged from 52.53% to 0.97%, with the magnitude of variation significantly greater than that of the diploid group. In terms of the range of variation, the triploid progeny line showed greater pollen activity and pollen volume, which is important as these superior strains are important breeding materials for parent selection studies. At the same time, pollen-free offspring appeared in all progeny populations, including five pollen-free strains in triploid progeny, which can be used as special polyploid germplasm material.

Parental dominance in the pollen mass of triploid hybrid progeny was negative and showed a negative genetic trend with −47.39% and −4.07% in 2022–2023, respectively. However, some hyper-parental progeny appeared in the triploid progeny group for pollen traits, with 24.49% hyper-parental dominance for pollen activity in 2022 and 33.33% hyper-parental dominance for pollen quantity in 2023. Pollen activity and pollen quantity in 2022–2023 were 68.85% and 40.74% for ultra-low parental dominance, respectively. The progeny showed a tendency of convergence to medium-low inheritance and was more inclined to parentage inheritance.

### 2.4. Characterization of the Distribution of Natural 2n Pollen in Jujube Date Triploid Hybrid Progeny Clusters

The distribution characteristics of the naturally produced 2n pollen in the parents and hybrid progeny were compared to analyze the type of naturally produced pollen in the triploid progeny (Table 7). The triploid progeny were found to produce a higher rate of 2n pollen than diploid progeny, producing 22.52% and 7.64% of the 2n pollen in 2022–2023, respectively. The triploid progeny produced mainly n pollen. A total of 74.16% and 89.13% of n pollen, 1.75.00% and 3.00% of shrunk pollen, and 1.57% and 0.23% of 3n or more pollen types were produced in 2022–2023, respectively. The hybrid progeny all showed a small amount of shrunk pollen, which varied by year.

The range and magnitude of variation in the type and amount of pollen naturally produced by the hybrid progeny were measured (Table 8, Figure 7). Triploid cross progeny were found to produce a greater proportion of 2n pollen, with 52.16% and 28.95% in 2022–2023, respectively, which informed the screening of the superior lines for the extreme natural production of 2n pollen. Meanwhile, the coefficient of variation for 2n pollen production by the triploid progeny was higher, with 51.47% and 102.36% in both years, respectively. The triploid offspring produced a maximum of 94. 00% and 99.13% of n pollen for the two years, respectively. A maximum of 35.29% of 3n or more pollen was produced in 2022. There was a large difference between the two years in 2n pollen rates in the triploid progeny. The extreme values of the 2n pollen rate in 2022–2023 were 52.16% and 28.95%, and the minimal values were 5% and 0%, respectively. In 2022, 35.29% of the 3n pollen was produced, and it is speculated that the rate of 2n vs. 3n and above pollen may be more genetically or environmentally influenced, resulting in the erratic production of pollen in both years.

### 2.5. The High 2n Pollen Line Germplasm Screening

For two consecutive years, lines that naturally produced 2n pollen in the triploid progeny of the cross were counted. Six superior triploid lines with high 2n pollen production were selected (Table 9). The 2n pollen rate ranged from 7.30% to 12.26%. Among them, Q20, Q36, and Q41 had higher 2n pollen rates of 12.26%, 9.17%, and 8.48%, respectively. The pollen activity ranged from 19.61% to 30.70% with a higher pollen activity of 29.55%, 30.70%, and 26.00% in Q35, Q41, and Q92, respectively. These screened jujube date triploid hybrid progenies that naturally produce a high rate of 2n pollen are important germplasm resources for ploidy breeding.

## 3. Discussion

### 3.1. Analysis of Abnormal Meiotic Behavior in Triploids

This study systematically analyzed the correspondence between flower bud developmental stages and meiotic progression, revealing significant correlations between the flower bud size, coloration, calyx fissure degree, and microspore mother cell meiosis phases [25]. These findings align with previous reports by Lü Ye [23] and Kang Xiangyang [26] in various plant systems (e.g., *Ziziphus jujuba* Mill. and *Popolus tomentosa carr.*), establishing morphological indicators for characterizing meiosis in jujube.

The critical period for 2n gamete formation occurs during meiosis I [27]. Cytological observations of the triploid germplasm Q161 demonstrated pronounced chromosomal abnormalities during meiosis I, including lagging chromosomes, chromosome bridges, and aberrant spindle configurations. These defects mirrored observations in triploid watermelon (*Citrullus lanatus*), where the lagging chromosomes of meiocytes led to unbalanced gametes [28], and in *Arabidopsis*, where spindle misorientation caused the production of about 60% dyads and 30% triploid offspring in male meiocytes [29]. Even with normal meiosis I completion, abnormal cytokinesis during telophase II could generate diploid (2n) pollen through dyad/triad formation [30]. Notably, our investigation identified defective cytoplasmic segregation during meiosis II, resulting in abnormal tetrads consistent with findings by Shao F et al. [19,31,32].

Analysis of the abnormal tetrads revealed that the abnormal tetrads formed by Q161 were mainly characterized by multifractal structures with abnormal cytoplasmic distribution (including trichomes and pentamers) and atypical tetrads carrying micronuclei. These structural anomalies showed significant correlation with drastically reduced pollen viability (4.0–4.67%). Similar phenomena have been documented in jujube [21] and *Asparagus officinalis* [33]. It is noteworthy that although about 9.00% of the abnormal tetrads carried micronuclei, it was not investigated whether there was a direct correlation between micronucleus formation and the abortive phenotypes such as the dryness and deformity of pollen grains in the progeny. This discrepancy may reflect differential tolerance to chromosomal loss: in Arabidopsis, micronuclei activate autophagy to eliminate defective microspores [34], whereas jujube might lack such compensatory mechanisms, allowing micronucleated grains to persist but remain nonviable. Building on the genetic regulatory mechanisms of pollen development elucidated in model systems like *Oryza sativa* [35] and *Arabidopsis thaliana* [36], combined with our phenotypic surveys and cytological evidence, we propose that the primary cause of pollen abortion in the triploid Q161 stems from inherent genomic instability (e.g., triploid-induced mispairing) rather than secondary cytological phenomena like micronuclei formation.

### 3.2. Chromosome Number and Karyotype Analysis of Q161

Chinese jujube chromosomes are generally 1–4 μm in length [37]. The chromosome lengths of Q161 (0.828 μm to 3.058 μm) confirmed its genomic stability within the *Ziziphus* lineage. Chinese jujube is typically diploid with 24 chromosomes. Previous studies identified 36 chromosomes in the natural triploid ‘Zanhuang Dazao’ and 48 chromosomes in the tetraploid ‘Chenguang’ [38]. In this study, the conventional squash preparation of pollen mother cells confirmed that Q161 had 36 chromosomes, verifying its triploid status, which was consistent with previous reports on spontaneous polyploidization in jujube [39].

Karyotypic symmetry and karyotypic features are commonly used to assess kinship and phylogenetic relationships between species, which is a critical parameter for phylogenetic inference. Jujube is categorized into 2A, 3A, and 2B types based on Stebbins’ classification [40]. Qu Zezhou et al. [41] reported that the triploid ‘Zanhuang Dazao’ has a 2B-type karyotype, while the cultivated ‘Xiangfen Yuanzao’ and wild Cisuanzao exhibit a 3A-type with high symmetry. Chen Yongli [42] found a 2A-type in the ‘Jinsixiaozao’ variety. Analysis revealed that Q161 contains 24 submetacentric chromosomes (sm) and 12 metacentric chromosomes (m), with the formula 2n = 3x = 36 = 12m + 24sm (4SAT). According to Stebbins’ classification [40], Q161 has a 3B-type karyotype. Compared with ‘Zanhuang Dazao’ (2B-type) [38], the triploid Q161 (3B-type) displayed greater karyotype heterogeneity, indicating more advanced chromosomal structural evolution. From a phylogenetic perspective, the karyotype symmetry of *Ziziphus* species correlates with their evolutionary status, suggesting that polyploidization may accelerate speciation through chromosomal rearrangements [38]. Notably, the predominance of sm chromosomes in the karyotype may relate to aneuploid variation. The sm chromosomes in jujube often harbor repetitive sequences and transposable elements, as revealed by recent gapless genome assemblies, which could promote non-homologous recombination and karyotype diversification [43]. Existing studies demonstrate that submetacentric chromosomes are more prone to breakage and fusion events, as demonstrated in oil tea [44] and *Brassicaceae* [45]. This structural vulnerability may explain the frequent lagging chromosomes and micronuclei observed in Q161’s meiocytes. The karyotype and chromosomal characteristics of Q161 determined in this study provide critical references for future research on polyploid formation mechanisms and chromosomal evolution in jujube.

From an evolutionary perspective, the 3B-type karyotype of Q161 signifies a transitional state towards higher ploidy levels. The karyotypic features of Q161 also have practical implications for breeding. While sm-rich karyotypes correlate with reduced pollen viability, they may simultaneously enhance heterosis via allelic diversity, as observed in triploid watermelon (*Citrullus lanatus*) [46,47] Additionally, the 3B-type’s structural complexity could buffer against aneuploidy-related fitness costs, a phenomenon documented in polyploid *Triticum aestivum* [48]. Future studies should integrate fluorescence in situ hybridization (FISH) to map satellite DNA and rDNA loci, thereby refining Q161’s karyotype evolution model.

### 3.3. Analysis of Natural 2n Pollen Occurrence in Triploid Hybrid Progeny

Polyploid sexual hybridization serves as a principal approach for crop germplasm innovation, achieving efficient chromosomal recombination through the fusion of gametes with different ploidy levels [44,49]. In oil tea camellia (*Camellia oleifera Abel.*), interspecific hybridization mediated by 2n pollen has successfully generated a cold-resistant tetraploid germplasm with 32% enhanced tolerance [50]. Wheat (*Triticum aestivum* L.) breeding has incorporated 2n pollen to precisely improve the genetic characteristics of wheat and increase the protein content and grain content [51]. These successes demonstrate that integrating pollen viability control techniques (e.g., cryopreservation) with cellular engineering methods (e.g., nuclear transplantation) can significantly enhance hybrid breeding efficiency [52], establishing paradigms for horticultural crop industrialization (e.g., polyploid rose cut-flower cultivar development). Polyploid hybrid crops like rice [53] and potato [54] obtained through 2n pollen hybridization exhibit superior characteristics, demonstrating both heterosis and enhanced stress resistance via genomic dosage effects [55]. In fruit trees, triploid (2n × n→3x) or tetraploid (2n × 2n→4x) germplasms can be efficiently created through specific hybridization modes [56]. Although natural 2n pollen has been found in apple (*Malus pumila* Mill., 0.014–1.71%), grape (*Vitis vinifera* L., 0.015–5.85%) and pear (*Pyrus* spp., 0.02–0.38%) [57], its frequency generally remains below 6%. This study identified six triploid hybrid progenies with stable 2n pollen occurrence rates of 7.30% to 12.26%. Individual lines were able to produce up to 52.16% of the 2n pollen, but it was unstable. We attribute the elevated 2n pollen formation in Xinjiang accessions to high-temperature exposure during the floral phase (particularly during flower bud differentiation and calyx development stages), aligning with Li Z et al.’s model where temperatures ≥ 35 °C disrupt microtubule dynamics, promoting dyad formation via spindle disassembly [16,58], and stimulate 2n pollen production in *Populus* [11]. These results confirm the environmental regulation of 2n pollen formation. The six high-yielding accessions (>7% occurrence rate) with stable 2n pollen production could serve as core parental materials for Chinese jujube polyploid breeding, though their pollen fertility requires further investigation.

The triploid progeny group exhibited significantly higher mean 2n pollen occurrence rates (22.52 ± 11.59%, 7.64 ± 7.82%) compared to the diploid controls (11.10 ± 10.47%, 2.44 ± 2.35%) (*p* < 0.005), strongly supporting the “positive ploidy–2n pollen correlation” principle [59]. Further analysis revealed a 51.47–102.36% coefficient of variation in 2n pollen production between the 2022–2023 years. This aligns with reports in *Triticum aestivum*, where environmental and genetic interactions caused 2n pollen production variability exceeding 80% [60]. Pollen quantity and viability assessments confirmed low yield and instability in 2n pollen production, aligning with previous reports in *Populus* [11]. Future priorities include the following: (1) identifying germplasms with high fertility and compatibility for polyploid breeding, (2) developing efficient pollen culture protocols, (3) exploring novel induction technologies to improve 2n pollen yield, and (4) extending 2n pollen applications to broader crop genetic improvement programs.

### 3.4. Comparative Analysis of Pollen Characteristics and Genetic Variation in Triploid Hybrid Progeny

Pollen viability, as a polygenically controlled quantitative trait, exhibits phenotypic variation governed by both additive and dominant genetic effects [17]. This study systematically compared pollen characteristics across the Ziziphus progenies of different ploidy levels to elucidate genetic variation patterns, providing theoretical foundations for parental selection in hybridization. Results demonstrated significant environmental influences on pollen traits, with marked phenotypic discrepancies between biennial datasets (the 2022–2023 years). The polyploid hybrid progenies showed extensive variation in pollen viability and production quantity. Specifically, the coefficients of variation for pollen production reached 51.47% and 102.36% across two consecutive years (2022–2023 years), indicating substantial segregation and predominant non-additive effects, consistent with findings by Yiling Pan et al. in jujube [61].

Comparative analysis between diploid and triploid progenies revealed that the triploid progeny groups exhibited more pronounced transgressive segregation, while their pollen morphological traits demonstrated a centralizing inheritance pattern with a tendency toward lower phenotypic values compared to diploid counterparts. In 2022, no statistically significant differences were observed in pollen quantity and viability between these groups, aligning with findings reported by Jinxia Liu [62]. However, our 2023 investigation revealed statistically significant differences (*p* < 0. 05): the pollen activity and quantity of triploid progeny were lower than that of the diploid control, a phenomenon further corroborated in sexual hybridization trials within *Citrus* spp. [63]. Meanwhile, the transcriptomic profiling of stress-responsive genes (e.g., HSP90, ARF) during microgametogenesis could disentangle genetic versus environmental contributions to pollen defects, as pioneered in cotton (*Gossypium* Linn.) [64]. Additionally, adopting high-throughput phenotyping tools, such as automated pollen viability recognition and AI-based viability assays [65], would enhance the resolution in quantifying subtle trait variations.

## 4. Conclusions

This study demonstrates a definitive correlation between the flower bud developmental stages and the meiotic process in the triploid germplasm. Cytological observations revealed multiple aberrant cytogenetic behaviors during meiosis, including chromosomal lagging, unequal distribution, and abnormal spindle fiber traction. These irregularities culminated in the formation of eight distinct types of aberrant tetrads during the tetrad stage, subsequently leading to 2n pollen formation. Furthermore, the 2n pollen grains exhibited distinctive morphological characteristics, manifested as a significantly larger size (≥1.5× the diameter of normal pollen), prominent exine ornamentation with intensified reticulation patterns, and a germination pore that was prominent and verrucose. The hybrid progeny naturally produced 22.52% and 7.64% of the 2n pollen in 2022–2023, respectively. Six triploid germplasm accessions with naturally high 2n pollen production rates (≥5.96%) were identified. The results of the observation of the meiotic behavior of the triploid germplasm of the Chinese jujube and the formation of a 2n-pollen-producing population provide important theoretical references for the study of the 2n gametes of *Ziziphus* and polyploid breeding.

## 5. Materials and Methods

### 5.1. Materials

In 2016, a hybrid population was obtained by crossbreeding the diploid ‘Dongzao’ (DZ) strain as the mother and tetraploid ‘Chenguang’ (CG) strain as the father, pollinated by honeybees [66], and the pollen of this hybrid population was used as the material for this experiment. The seeds were sown in 2017 in Greenhouse No. 4 at the Horticultural Experiment Station of Tarim University. The scions were collected until 2021 and highly grafted in the jujube date germplasm nursery of the 12th Regiment of the 1st Division in Alar City, Xinjiang, China (80°5′–81.97′ E, 40°37′–40°95′ N; altitude, 110 m). The grafting rootstock was the 6-year-old ‘Huizao jujube’. The orchard row spacing was 1 m × 2 m, the water and fertilizer germplasm resource garden was sufficient for growth, and the management level was kept consistent. A total of 72 triploid offspring and 36 diploid offspring were identified from the obtained hybrid population [25].

The parents and progeny were sampled during May–June 2022–2023 from 9:30 to 11:30 a.m. Thirty flower buds in the middle of the jujube that were in full bloom and about to open were picked for the pollen counts. Ten freshly opened flower buds were picked and used for the pollen activity assay. Ninety flower buds of the superior line Q161 of the cross progeny were collected at the peak of meiosis of the jujube flowers (present order and early bud-yellow stage) and used for meiosis observation. Fifteen flower buds were randomly selected from the middle of the secondary branches at full bloom when the buds had expanded to a bell shape, had not exhibited nectar discs or pollen, and the flowers were about to open (large bud stage). The period of collection was recorded, and the samples were placed in an insulated box and brought back to the laboratory for the determination of relevant test traits.

### 5.2. Methods

#### 5.2.1. Observations of Meiosis in Microspore Mother Cells

The flower buds of the retrieved hybrid progeny superior line Q161 at different flowering stages (30 collected separately) were observed according to the method of Lv Ye et al. [22] with slight modifications, and the modified method was as follows: the collected flower buds were placed in Carnot’s fixative (anhydrous ethanol:glacial acetic acid = 3:1) after determining the length of the flower buds using vernier calipers for 24 h. The buds were rinsed sequentially using a 95% → 85% → 70% concentration of alcohol, then placed in 70% ethanol solution and stored in a refrigerator at 4 °C for the spare time. Sampling was continued until the anthers were mature and dispersing pollen. For microscopic examination, flower buds were washed with distilled water to remove ethanol placed on filter paper to absorb the excess liquid. The pollen grains in the flowering anthers were peeled off with a dissecting needle and forceps placed on clean slides, stained and pressed with a propionic acid–iron hematoxylin–hydrated trichloroacetaldehyde (PIHCH) staining solution for 3–8 min, and pressed again. The meiotic process of the Q161 samples was observed using an OLYMPUS light microscope (BX41; Olympus, Tokyo, Japan) and photographed with the imaging system MDC200 (LY-HPCCD) and Imagine Analysis System 10.0.

#### 5.2.2. Determination of Pollen Traits

Pollen activity assay: The I_2_-KI assay was used [67], where freshly opened buds were stripped of the anthers and placed on slides, and then 2–3 drops of iodine–potassium iodide (I_2_-KI) solution was placed on the slides. The pollen was gently clamped with tweezers for 8–12 strokes, and the anthers were clamped out and stained for 5–8 min. After pressing the slices, the slides were observed in an electron microscope (Bn51 Olympus) with a 20× objective. Five fields of view were observed for each coverslip and six replicates were averaged. Pollen grains with pollen activity are dark red or dark brown and the inactive pollen grains are light red or light brown.

Pollen activity formula: Pollen activity (%) = (number of dark pollen/total number of pollen) × 100%.

Pollen volume determination: A hematocrit plate was used [25], and the flower buds that were about to bloom, stripped of 90 anthers, with 30 anthers in one replicate, were placed in 2 mL centrifuge tubes. The anthers were left for 3–5 days, dried naturally so that the anthers were completely opened, and then 2% sodium hexametaphosphate solution was added to make the volume up to 2 mL. After shaking well using the vortexer, 20 μL of the pollen suspension was aspirated on the hemocyte counting plate with a pipette gun and observed with an electron microscope (Bn51 Olympus) with a 10× objective lens; 4 fields of view were observed for each tube of the pollen suspension, and the average was taken from 3 repetitions. The amount of pollen in 400 squares on the counting board was taken as the number of single pollen grains as the amount of pollen for that test material.

Pollen count formula: Pollen count (grains^−1^) = (total pollen count in 400 squares × 2000)/30

#### 5.2.3. Determination of Pollen Type

Twenty randomly selected fields of view from the pollen activity trait assay performed in Section 5.2.2 were used to measure 300 pollen diameters. The increase in pollen DNA content was accompanied by a corresponding increase in pollen diameter, and the gigantization of pollen implied the production of 2n pollen [22]. The 2n pollen identification in this experiment was standardized by Peng Bo [68] for the jujube date 2n pollen being 1.3 times the n pollen. The test subjects of this study were the progeny clusters produced by crossing the diploid ‘Dongzhao’ with the tetraploid ‘Chenguang’, in which ‘Dongzhao’ produced n pollen and ‘Chenguang’ produced 2n pollen. The measured pollen grains were categorized as deflated pollen, n pollen, 2n pollen, 3n and above pollen, and this grading criteria are shown in Table 10.

#### 5.2.4. Scanning Electron Microscopy of Pollen

The large bud-stage flower buds in the middle of the secondary branches, which were randomly collected in the previous period, were carefully stripped of the anthers with clean forceps; the anthers were placed in 2 mL centrifuge tubes and stored indoors at room temperature to allow them to dry. The dried pollen was flicked onto a sample tray with double-sided adhesive, sprayed by a JFC-1600 ion sputterer under vacuum for 80 s, and placed under an APREO-S scanning electron microscope (SEM) (Thermo Fisher, Waltham, MA, USA) for observation. A typical field of view was selected to be photographed. Scanning electron microscopy of the pollen was performed at the Analysis and Testing Center at Tarim University.

### 5.3. Data Processing

Data were recorded and organized in Excel 2016 and processed for plotting and analysis using SPSS 27.0 Origin 2024.

## Figures and Tables

**Table 1:** The corresponding relationship between the meiosis period of the triploid germplasm and the external morphology of flower buds.

Microspore Blastogenesis	Range of Flower Bud Diameters/mm	Morphological Characteristics of Flower Buds
Pollen mother cell period	1.3–2.1	Dark green; pollen outline gradually clearing; sepal slits gradually evident
Preliminary I	2.1–2.8	Dark green to yellowish green; slightly enlarged; sepal slits gradually evident
Terminal II	2.8–3.1	Yellowish-green; volume expansion evident; sepal slits largely evident
Mononuclear pollen	3.1–3.9	Yellowish-green; reaching maximum size, at large bud stage; sepals about to unfurl

**Table 2:** Morphological patterns of the meiotic tetrads in the selected triploid elite line Q161 of jujube.

Type	Quantity/pc	Proportion/%
Dyad	14	4.60%
Trisomy	23	7.50%
Trisomy + micronucleus	6	1.90%
Tetrads	237	77.20%
Tetrads + micronuclei	21	6.80%
Pentads	4	1.30%
Pentads + micronuclei	1	0.30%
Hexads	1	0.30%

**Table 3:** Triploid elite line Q161 chromosome length.

Serial No.	1	2	3	4	5	6	7	8	9	10	11	12
Length (μm)	2.430	2.94	1.765	1.625	1.408	1.188	1.801	1.487	0.828	1.411	1.241	1.908
Serial No.	13	14	15	16	17	18	19	20	21	22	23	24
Length (μm)	2.789	1.885	1.252	2.266	2.056	1.160	1.503	2.331	1.686	1.916	1.173	1.687
Serial No.	25	26	27	28	29	30	31	32	33	34	35	36
Length (μm)	3.059	1.877	2.735	0.941	0.998	1.603	1.955	1.486	1.213	1.206	1.211	2.74

**Table 4:** Pollen traits and pollen frequency distribution of jujube triploid superior line Q161.

Pollen Traits	2022	2023
Pollen activity (%)	39.81 ± 3.30	22.10 ± 6.39
Pollen amount (%)	461.11 ± 101.84	1522.22 ± 139.77
Pollen diameter (μm)	23.61 ± 4.05	22.40 ± 3.52
Shrunk pollen (%)	0.33	1.00
n pollen rate (%)	71.33	83.67
2n pollen rate (%)	4.00	4.67
3n pollen rate (%)	0.67	0.33

**Table 5:** Comparison of pollen traits between parents and two progeny groups.

Traits	Parents and Ploidy	Pollen Activity/%		Pollen Quantity/Grain	
		2022	2023	2022	2023
Mean ± SD	DZ (2X)	16.56 ± 8.90 b	16.89 ± 3.17 a	2427.78 ± 233.53 a	1155.56 ± 108.44 ab
	CG (4X)	38.60 ± 13.17 b	33.68 ± 4. 46 b	1694.45 ± 353.29 b	1305.56 ± 635.16 b
	Triploid progeny (3X)	30.45 ± 9.04 a	23.83 ± 4.62 c	942.53 ± 763.14 b	1438.96 ± 755.84 ab
	Diploid progeny (2X)	33.66 ± 5.73 a	29.21 ± 5.38 d	925.37 ± 551.20 b	2118.60 ± 823.72 a

Note: *t*-tests of independence for the parent and two offspring cohorts were performed; Letters (a,b,c,d) in the table represent significance at *p* < 0.05, the difference is considered significant.

**Table 6:** Genetic variation analysis of pollen traits in two progeny groups.

Traits	Ploidy	Years	CV/%	Variation Range	*MPH*/%	*RH*/%	*RL*/%
Pollen activity/%	Triploid progeny	2022	29.69	15.13–46.90	10.41	24.49	4.08
		2023	19.39	13.40–35.52	−5.77	2.13	8.51
	Diploid progeny	2022	17.02	22.7–44.53	22.04	30.77	0
		2023	18.42	23.23–40.41	15.50	21.74	0
Pollen quantity/grain	Triploid progeny	2022	80.97	0.00–37.50	−47.39	4.92	68. 85
		2023	52.53	0.00–3377.78	−4.07	33.33	40.74
	Diploid progeny	2022	59.57	16.67–1888.89	−48.35	0	60.00
		2023	38.88	0.00–4416.67	41.24	75.00	12.50

**Table 7:** Analysis of the proportion of pollen types from the parents and different ploidy hybrids.

	Years		Shrunk Rate/%	n Pollen Rate/%	2n Pollen Rate/%	3n and Above Pollen Rate/%
Parents	2022	DZ (2X)	2.00	92.67	5.33	0.00
		CG (4X)	0.00	50.00	49.00	1.00
	2023	DZ (2X)	9.33	89.00	1.67	0.00
		CG (4X)	0.00	50.67	46.67	2.67
Hybrid progeny	2022	Triploid progeny	1.75 ± 2.12 *	74.16 ± 14.10 **	22.52 ± 11.59 **	1.57 ± 4.88
		Diploid progeny	3.06 ± 2.49	85.64 ± 10.35	11.10 ± 10.47	0.19 ± 0.67
	2023	Triploid progeny	3.00 ± 4.06	89.13 ± 7.70 **	7.64 ± 7.82 **	0.23 ± 0.48 **
		Diploid progeny	2.17 ± 1.60	95.39 ± 2.48	2.44 ± 2.35	0.00 ± 0.00

Note: *t*-tests for independence were performed for two different ploidy offspring in the same year. *: At the 0.05 level (two-tailed), the difference is considered significant. **: At the 0.01 level (two-tailed), the difference is considered significant.

**Table 8:** Variation in pollen types in different ploidy progeny.

Years	Type	Diploid			Triploid		
		Minimum Rate/%	Maximum Rate/%	CV/%	Minimum Rate/%	Maximum Rate/%	CV/%
2022	Shrunk pollen rate	0.00	11.82	81.37	0.00	10.85	121.14
	n pollen rate	47.91	96.07	12.09	12.55	94.00	19.01
	2n pollen rate	0.44	46.25	94.32	5.00	52.16	51.47
	3n and above pollen rate	0.00	2.92	352.63	0.00	35.29	310.83
2023	Shrunk pollen rate	0.00	6.33	73.73	0.00	17.67	135.33
	n pollen rate	91.33	98.67	2.60	69.17	99.13	8.64
	2n pollen rate	0.00	8.33	96.31	0.00	28.95	102.36
	3n and above pollen rate	0.00	0.00	0.00	0.00	2.64	208.70

**Table 9:** Triploid superior lines with high 2n pollen rates in hybrid offspring.

Name	2n Pollen Rate/%	Pollen Activity/%	Pollen Amount/Grain
Q20	12.26	19.61	1094.44
Q35	8.05	29.55	636.11
Q36	9.17	21.30	950.00
Q40	5.96	24.04	697.22
Q41	8.48	30.70	827.78
Q92	7.30	26.00	736.11

**Table 10:** Classification standard of parental pollen diameter.

Year	Pollen Diameter/μm							
	Parents				Pollen Type Grading Scale (P)			
		Mean	1.3 Times	1.5 Times	Shrunk Pollen	n Pollen	2n Pollen	3n and Above Pollen
2022	DZ	21.45	27.89	32.18	≤15.00	15.00 < P ≤ 25.99	25.99 < P ≤ 33.78	>33.78
	CG	25.99	33.78	38.98				
2023	DZ	20.14	26.19	30.21	≤15.00	15.00 < P ≤ 28.62	28.62 < P ≤ 37.21	>37.21
	CG	28.62	37.21	42.93				

## Data Availability

The data will be made available upon request.
